# Seasonal Trophic Shift of Littoral Consumers in Eutrophic Lake Taihu (China) Revealed by a Two-Source Mixing Model

**DOI:** 10.1100/tsw.2011.134

**Published:** 2011-07-28

**Authors:** Qiong Zhou, Ping Xie, Jun Xu, Xufang Liang, Jianhui Qin, Te Cao, Feizhou Chen

**Affiliations:** ^1^ Donghu Experimental Station of Lake Ecosystems, State Key Laboratory of Freshwater Ecology and Biotechnology of China, Institute of Hydrobiology, Chinese Academy of Sciences, Wuhan, China; ^2^ College of Fisheries, Huazhong Agricultural University, Wuhan, China; ^3^ State Key Laboratory of Lake Science and Environment, Nanjing Institute of Geography and Limnology, Chinese Academy of Sciences, Nanjing, China

**Keywords:** stable isotope, mixing model, trophic shift, benthic and planktonic energy sources, Lake Taihu

## Abstract

We evaluated the seasonal variation in the contributions of planktonic and benthic resources to 11 littoral predators in eutrophic Lake Taihu (China) from 2004 to 2005. Seasonal fluctuations in consumer σ^13^C and σ^15^N were attributed to the combined impacts of temporal variation in isotopic signatures of basal resources and the diet shift of fishes. Based on a two-end-member mixing model, all target consumers relied on energy sources from coupled benthic and planktonic pathways, but the predominant energy source for most species was highly variable across seasons, showing seasonal trophic shift of littoral consumers. Seasonality in energy mobilization of consumers focused on two aspects: (1) the species number of consumers that relied mainly on planktonic carbon showed the lowest values in the fall and the highest during spring/summer, and (2) most consumer species showed seasonal variation in the percentages of planktonic reliance. We concluded that seasonal trophic shifts of fishes and invertebrates were driven by phytoplankton production, but benthic resources were also important seasonally in supporting littoral consumers in Meiliang Bay. Energy mobilization of carnivorous fishes was more subject to the impact of resource availability than omnivorous species.

### INTRODUCTION

Energy flow through ecosystems is of fundamental importance for understanding the function of lake ecosystems. Mobilization and transfer of energy within the food web sets the limit for production at higher trophic levels, and the presence of several diverse energy flow pathways may affect ecosystem properties, such as food web dynamics and nutrient cycling[1]. Research that evaluates lake energy flow has largely focused on pelagic food webs. Pelagic energy mobilization derives from photosynthesis and from phytoplankton growing on allochthonous organic carbon[2,3,4]. However, recent studies have shown that benthic resources are of great importance for lake food webs because fish communities may make considerable use of zoobenthos and periphyton[5,6,7,8]. Benthic and pelagic energy flows and processes can be coupled in a variety of ways[7,9], including cross-habitat foraging by fishes. Many fishes exhibit flexible feeding habits and undergo diet shifts that deviate from their presumed food chain[10]. Often, carnivorous fishes forage broadly upon pelagic and benthic fishes, and thereby link the pelagic and benthic portions of food webs[11,12]. Hence, benthic–pelagic coupling has been a focus of ecologists and has been considered among the most important habitat coupling in lake food webs[8,13].

Lake Taihu, with an area of 2428 $km^2$ and a mean water depth of 1.9 m, is the third largest freshwater lake in China. During the past decades, the lake has undergone a steady eutrophication, characterized by a regular occurrence of cyanobacterial surface blooms in the warm seasons of each year[14]. Meiliang Bay (31°31′-325′N, 120°09′-340′E), located in the northern part of Lake Taihu, accommodates municipal and industrial wastewater from Wuxi City (Jiangsu Province), and acts as the principal water resource and recreational spot for Wuxi City. In recent decades, due to annual outbreaks of *Microcystis* blooms, this region has been one of the most hypereutrophic parts of Lake Taihu[15]. Increasing attention has been paid to this shallow lake regarding the eutrophication and cyanobacterial blooms[15,16,17,18,19,20], whereas very few researchers have addressed energy mobilization and transfer in the food web. Although littoral fishes in Meiliang Bay are subsidized by pelagic production (phytoplankton) derived from algal blooms[15,18,19], aquatic plants and periphyton have covered the littoral zones to a certain extent due to the shallow water level (mean water depth: 2.17 m). Rooted macrophytes can obtain most of their nutrients through root uptake from sediments and periphyton often compete with planktonic algae for limiting nutrients such as phosphorus and nitrogen[21]. So, in Meiliang Bay, whether phytoplankton-based energy sources dominate the energy supply for littoral consumers year round is still unknown.

Naturally occurring stable isotopes of carbon ($\delta^{13}C$) and nitrogen ($\delta^{15}N$) in organisms provide a time-integrated measure of trophic positions and carbon flows through the food webs[22,23]. A consumer is typically enriched in $^{15}N$ by 3–4‰, relative to its diet[24,25], and hence $\delta^{15}N$ has been used to define trophic positions in food webs[26]. The stable carbon isotope also produces a trophic enrichment (0–1‰) for clarifying carbon sources in aquatic ecosystems and tracing carbon flow from primary producers to top consumers[27,28]. Stable isotopes have the potential to simultaneously capture complex interactions and to track energy or mass flow through ecological communities[29,30,31]. One common application is to use stable isotope mixing models to quantify source contributions to a mixture. A case in point is the fractional proportion of food sources in animal diets. Linear mixing models based on stable isotope mass balance have long been used for this purpose[32,33]. The simplest mixing model includes two isotopic sources (two-source mixing model), and can be used to assess the importance of littoral and pelagic energy sources in supporting the food web of lakes[13,23]. Surface-grazing snails reflect the isotopic signature of the base of the littoral food web (periphyton- and macrophyte-derived energy source), whereas filter-feeding mussels tend to reflect the isotopic signature of the base of the pelagic food web (phytoplankton-derived energy source)[23]. Together, these organisms provide an accurate isotopic baseline in lake ecosystems[23].

Here we used the two-source mixing model to assess the contribution of planktonic and benthic energy sources to higher trophic level consumers, and to determine the potentially seasonal pattern of resource use for the fish community in the littoral zones of Meiliang Bay.

## MATERIALS AND METHODS

### Sampling

The basic morphometrical and limnological characteristics of sampling sites in the present study are shown in Table 1. Consumer species, including fishes and invertebrates, were collected seasonally from the littoral zone (approximately 100 m from the shore, average water depth = 1.2 m) of Meiliang Bay (Fig. 1). We collected fish and invertebrate samples at 3-month intervals. Adult individuals were selected for fishes and invertebrates due to a potentially ontogenic diet shift. The sampling dates were October 10–13 (fall) in 2004 and January 13–15 (winter), April 11–13 (spring), and August 9–12 (summer) in 2005. Generally, three to five individuals of each species were collected due to resource availability at each sampling date. Fish and freshwater shrimp, *Macrobrachium nipponense*, were captured using a net (mesh size = 5 mm) named Yuduan, set at a place 100 m from the bank of Meiliang Bay. Zoobenthos (mussel, snail, and Chironomid larvae, etc.) were obtained from sediment approximately 15-cm deep by van Veen grabs (0.1 $m^2$) and sieved using a 1.0-mm screen. Zooplankton were sampled through a plankton net with a mesh size of 112 μm, then washed and kept in distilled water for about 2 h to allow for gut clearance, in order to eliminate bias in the stable isotope signature of the organism due to items in the recent diet. Sampled fishes and invertebrates were kept in an icebox and brought back to the laboratory for subsequent treatments.

**TABLE 1**
**The Average Values and Ranges of Selected Morphometrical and Limnological Characteristics in Meiliang Bay (Lake Taihu) from 2004 to 2005[34,55,56]**

| Parameter | Average | Range |
|---|---|---|
| Surface area ($km^2$) | 125 | nd |
| Storage capability ($m^3$) | $2.7 \times 10^8$ | nd |
| Water depth (m) | 2.17 | 1.00–3.43 |
| Temperature (°C) | 18.36 | 3.8–31.6 |
| Dissolved oxygen (mg/L) | 7.65 | 1.23–14.60 |
| Secchi depth (cm) | 37 | 10–-90 |
| pH | 8.02 | 7.25–-8.77 |
| Conductivity (μs/cm) | 581.7 | 415–1100 |
| Total algal biomass (mg/L) | 3.08 | 0.047–19.05 |
| Chlorophyll *a* (μg/L) | 22.15 | 2.68–168.47 |
| TN (mg/L) | 5.41 | 1.55–13.56 |
| TP (mg/L) | 0.16 | 0.043–0.46 |
| $NH_4$-N (mg/L) | 2.28 | 0.02–9.05 |
| $NO_3$-N (mg/L) | 1.02 | 0.08–2.96 |
| COD (mg/L) | 5.42 | 2.95–10.44 |
| $BOD_5$ (mg/L) | 3.88 | 0.12–10.26 |

TN: total nitrogen; TP: total phosphorus; “nd”: no data.

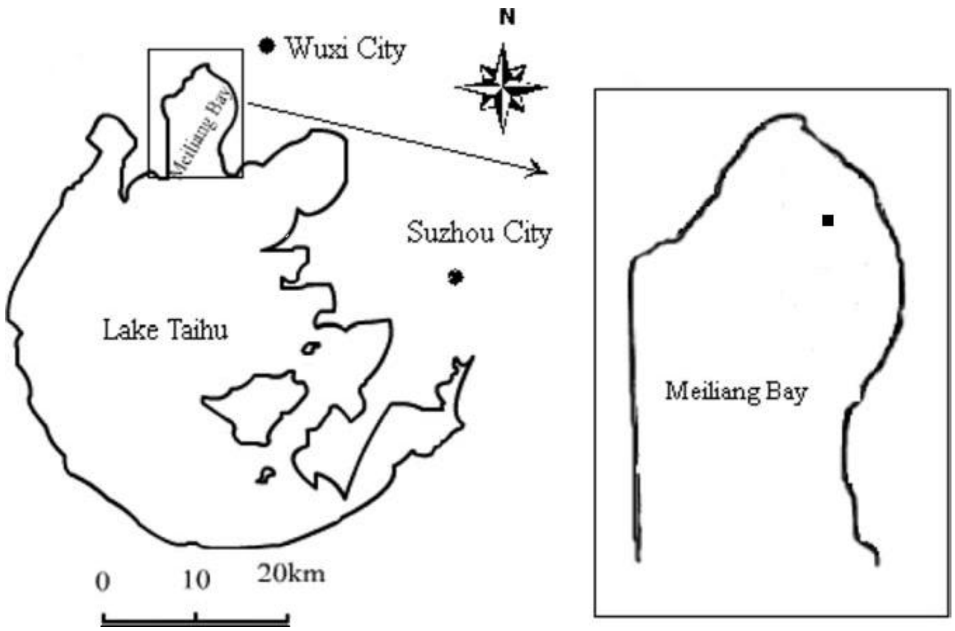

**FIGURE 1.** The sketch map of Lake Taihu and sampling station of Meiliang Bay.

Integrated water was collected from the surface and near the bottom layers using a 5-L modified Patalas bottle sampler. Phytoplankton samples were concentrated to 50 mL by sedimentation and preserved with Lugol's iodine solution immediately after sampling. Phytoplankton cells were counted under a microscope and sized to derive volumes from appropriate geometric shapes. Biomass (wet weight) was calculated assuming a wet weight density of 1 g/cm$^3$[34].

### Laboratory Treatment and Stable Isotope Analyses

Dorsal muscle tissues were anatomized from each fish because this tissue has been shown to be representative of the overall stable isotope signature of fish[35]. Whole small fish exhibited consistent fractionation with respect to diet and were used for stable isotope analyses instead of muscle tissue[36]. Abdomen muscle was determined for freshwater shrimp. For snails and mussels, only the foot muscle was used for stable isotope analysis because shell material shows enriched $^{13}$C and does not reflect the $\delta^{13}$C signatures of the food sources consumed[37]. Muscle tissues and zooplankton samples were roasted at 60°C to constant weight and ground to a fine powder using a mortar and pestle for $\delta^{13}$C analysis. Samples were analyzed with a Carlo Erba EA-1110 elemental analyzer accompanied by a Finnigan Delta Plus (Thermo Scientific, Waltham, MA, USA) continuous flow isotope ratio mass spectrometer (CF-IRMS). Isotopic ratios were expressed relative to international standards (Pee Dee Belemnite for carbon). Delta values were defined as: $\delta R = [(X_{sample} - X_{standard}) / X_{standard}] \times 10^3$ [‰], where R = $^{13}$C and X is the corresponding ratio $^{13}C/^{12}C$. Analytical deviations for replicates were <0.2 and 0.3‰ for carbon and nitrogen, respectively.

### Two-Source Mixing Model

Due to the relatively small surface area (125 km$^2$) and shallow water depth (average 2.17 m) of Meiliang Bay, there was no obvious borderline between the pelagic and littoral zones. Thus, we attempted to assess the contribution of the planktonic and benthic energy source to littoral consumers using a two-source mixing model: percent planktonic = ($\delta^{13}C_{consumer}$-$\delta^{13}C_{benthic}$) / ($\delta^{13}C_{planktonic}$-$\delta^{13}C_{benthic}$), which was proposed by Phillips[33] and modified from Vander Zanden and Vadeboncoeur[13]. Here we assumed that there is no trophic enrichment in $\delta^{13}C$ (trophic fractionation = 0‰) and that mixing is linear[13,23]. If the $\delta^{13}C$ value of consumers fell beyond either end member in the mixing model (indicating >100% planktonic reliance or <0% planktonic reliance), values were set at either 100 or 0%. In our study, zooplankton samples were not available year round, so snails (*Bellamya aeruginosa*) and mussels (*Cristaria plicata*) served as baseline indicators (trophic position = 2) of, respectively, benthic and planktonic energy sources, following the methodology emphasized in previous studies[31,38,39].

### Statistical Analyses

Statistical tests were performed with SPSS software, version 11.5. One-way ANOVA analyses with species and season as the main factors were executed to investigate the differences in stable isotope ratios of each species between four quarters (paired samples t-test). For each species, all individuals in fall, winter, spring, and summer respectively represented four different groups. Moreover, not less than two individuals were required for each group and at least three groups were requested for one-way ANOVA analyses. Furthermore, two-way ANOVA analysis with species and season as independent variables were performed to assess the effects of species and season on the proportional contributions of planktonic carbon to consumers

## RESULTS

### The $\delta^{13}C$ and $\delta^{15}N$ Signatures of Consumers

Eleven target consumer species are common inhabitants in the littoral zones of Meiliang Bay and can be captured year round. They are representative of different food habits and hence can reflect the impact of a phytoplankton-derived energy source on the littoral food web. Their total lengths and body weights are expressed as averages and ranges in Table 2.

**TABLE 2**
**Species Details of 11 Predominant Consumers**

| Name | Food Habit | Total Length (cm) | Body Weight (g) |
|---|---|---|---|
| *Cultrichthys erythropterus* | Carnivorous | 19.8–30.6 (25.4) | 85.7–278 (174.7) |
| *Coilia nasus* | Carnivorous | 18.0–24.9 (21.1) | 21.3–42.1 (29.5) |
| *Hemiculter leucisculus* | Omnivorous | 15.1–21.4 (18.3) | 19.2–37.6 (28.5) |
| *Toxabramis swinhonis* | Omnivorous | 10.2–14.0 (11.3) | 12.0–20.3 (15.1) |
| *Rhodeus ocellatus* | Herbivorous | 8.9–12.2 (10.3) | 10.6–20.2 (15.6) |
| *Abbottina rivularis* | Omnivorous | 9.5–12.8 (11.2) | 11.9–20.2 (15.0) |
| *Pseudorasbora parva* | Omnivorous | 7.7–11.8 (9.6) | 9.1–16.8 (13.3) |
| *Cyprinus carpio* | Omnivorous | 30.9–59.5 (48.2) | 344.7–2390 (799.0) |
| *Carassius auratus* | Omnivorous | 19.4–27.0 (23.5) | 86.6–316.5 (156.6) |
| Shrimp *Macrobrachium nipponense* | Omnivorous | — | 8.9–13.5 (11.6) |
| Chironomid larvae | Omnivorous | — | <1.0 |

"—" means unavailable. Classifications of food habits for consumers are according to Ni and Wu[57].

Stable carbon and nitrogen isotope signatures of consumers showed obvious interspecific differences at each season (Fig. 2). For $\delta^{13}C$, there were great differences of 6.3‰ (fall), 4.3‰ (winter), 7.0‰ (spring), and 3.8‰ (summer) between different consumers. Consumer $\delta^{15}N$ indicated differences of 8.3, 5.6, 6.2, and 5.6‰ in fall, winter, spring and summer, respectively.

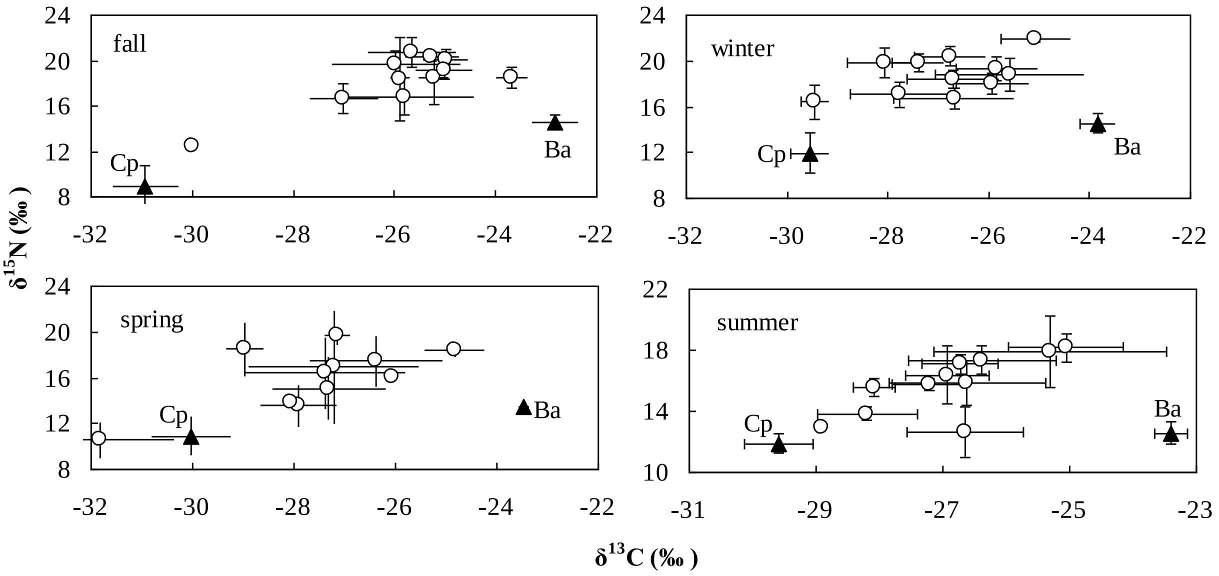

**FIGURE 2.** The $\delta^{15}N$ plotted against $\delta^{13}C$ of 11 consumer species (open circles) and two baseline indicators (solid triangles) in different seasons (fall, winter, spring, and summer). The $\delta^{13}C$ and $\delta^{15}N$ values were expressed by mean ± 1SD. Ba: snail *Bellamya aeruginosa*; Cp: mussel *Cristaria plicata*.

According to one-way ANOVA results, most of the 11 target consumers showed statistically significant between-quarter differences in $\delta^{13}C$ and/or $\delta^{15}N$ signatures, apart from *R. ocellatus* and *Cyprinus carpio* (Table 3). As baseline indicators, snails (*B. aeruginosa*) and mussels (*Cristaria plicata*) exhibited lesser variations of, respectively, 1.0 and 1.38‰ for $\delta^{13}C$ and 2.1 and 3.0‰ for $\delta^{15}N$ across seasons. Moreover, there was no statistically significant difference of isotopic signatures across seasons (Table 3).

### Contributions of Planktonic Carbon to Littoral Consumers

Based on the calculation of the two-end-member mixing model, most consumer species changed the main energy source between planktonic and benthic resources during the study period, apart from *R. ocellatus*, *Carassius auratus*, and Chironomid larvae (Fig. 3). *R. ocellatus* relied on planktonic carbon for 10.4, 30.5, 39.5, and 26.8% in fall, winter, spring, and summer, respectively, suggesting absolute reliance on the benthic energy source. Contrarily, both *C. auratus* and Chironomid larvae derived reliance of carbon primarily from a phytoplankton-derived energy source (Fig. 3). During the study period, omnivorous fishes, including *H. leucisculus*, *T. swinhonis*, *A. rivularis*, *P. parva*, *Cyprinus carpio*, and *M. nipponense*, switched resource use from benthic to planktonic carbon (Fig. 3). Dissimilarly, carnivorous fishes (*Cultrichthys erythropterus* and *Coilia nasus*) switched from benthic to planktonic reliance at first, and then from planktonic to benthic reliance (Fig. 3).

**TABLE 3**
**One-Way ANOVA Results Showing Seasonal Variations in Stable Isotope Ratios ($\delta^{13}C$ and $\delta^{15}N$) of 11 Target Consumers and Two Baseline Indicators with Species and Season as the Main Factors**

| | | $\delta^{13}C$ | | $\delta^{15}N$ | |
|---|---|---|---|---|---|
| **Species** | **d.f.** | ***F*** | ***p*** | ***F*** | ***p*** |
| *Cultrichthys erythropterus* | 3 | 3.746 | *0.045 | 3.189 | 0.052 |
| *Coilia nasus* | 3 | 16.251 | *0.000 | 4.251 | *0.022 |
| *Hemiculter leucisculus* | 3 | 3.986 | *0.040 | 6.425 | *0.007 |
| *Toxabramis swinhonis* | na | na | na | na | na |
| *Rhodeus ocellatus* | 2 | 1.943 | 0.199 | 0.312 | 0.739 |
| *Abbottina rivularis* | 3 | 24.740 | *0.000 | 45.070 | *0.000 |
| *Pseudorasbora parva* | 3 | 1.947 | 0.163 | 7.371 | *0.003 |
| *Cyprinus carpio* | 3 | 1.273 | 0.322 | 1.840 | 0.186 |
| *Carassius auratus* | 3 | 2.439 | 0.102 | 7.461 | *0.002 |
| Shrimp *Macrobrachium nipponense* | 3 | 8.725 | *0.001 | 7.775 | *0.002 |
| Chironomid larvae | 2 | 27.471 | *0.001 | 8.588 | *0.017 |
| Freshwater snail *Bellamya aeruginosa* | 3 | 1.016 | 0.375 | 1.562 | 0.224 |
| Mussel *Cristaria plicata* | 3 | 1.304 | 0.296 | 1.913 | 0.161 |

* Significant values with critical α level = 0.05.

"d.f." indicates degrees of freedom between groups; "na" indicates "not available" due to the limitation of statistical methods.

Planktonic reliance for littoral consumers showed a between-quarter difference (Table 4), especially between fall and other seasons. In the fall, most species relied primarily on benthic carbon, apart from the crucian *Carassius auratus* and Chironomid larvae, which derived 51.6 and 88.3%, respectively, of carbon from the planktonic energy source. However, in other seasons, most consumers greatly shifted their energy mobilization from benthic to planktonic carbon. Overall, two, six, eight, and eight species showed reliance on pelagic carbon in fall, winter, spring, and summer, respectively (Fig. 3).

### Biomass of Phytoplankton

As a potentially important energy source, biomass of phytoplankton in the lake water of the sampling site was evaluated. Phytoplankton were collected monthly from September 2004 to August 2005. Phytoplankton biomass ranged between 0.9 mg/L (November 2004) and 54.2 mg/L (August 2005). Averaged biomass of phytoplankton was 1.8, 1.6, 5.5, and 25.1 mg/L in fall, winter, spring, and summer, respectively.

### DISCUSSION

Our study has found significant seasonal variations in $\delta^{13}C$ and $\delta^{15}N$ signatures of common fishes and invertebrates in the littoral zones of Meiliang Bay. This might reflect that temporal variations in isotopic signatures of aquatic food resources (e.g., phytoplankton and benthic algae) transferred to higher trophic level predators via the food chain[18,40], but might also reflect that the diet shift resulted from food availability[19,41]. Based on gut content analysis, Ye[42] demonstrated that most fishes, such as *Cultrichthys erythropterus*, *Coilia nasus*, and *H. leucisculus*, fed on a wide variety of food and showed temporal variations

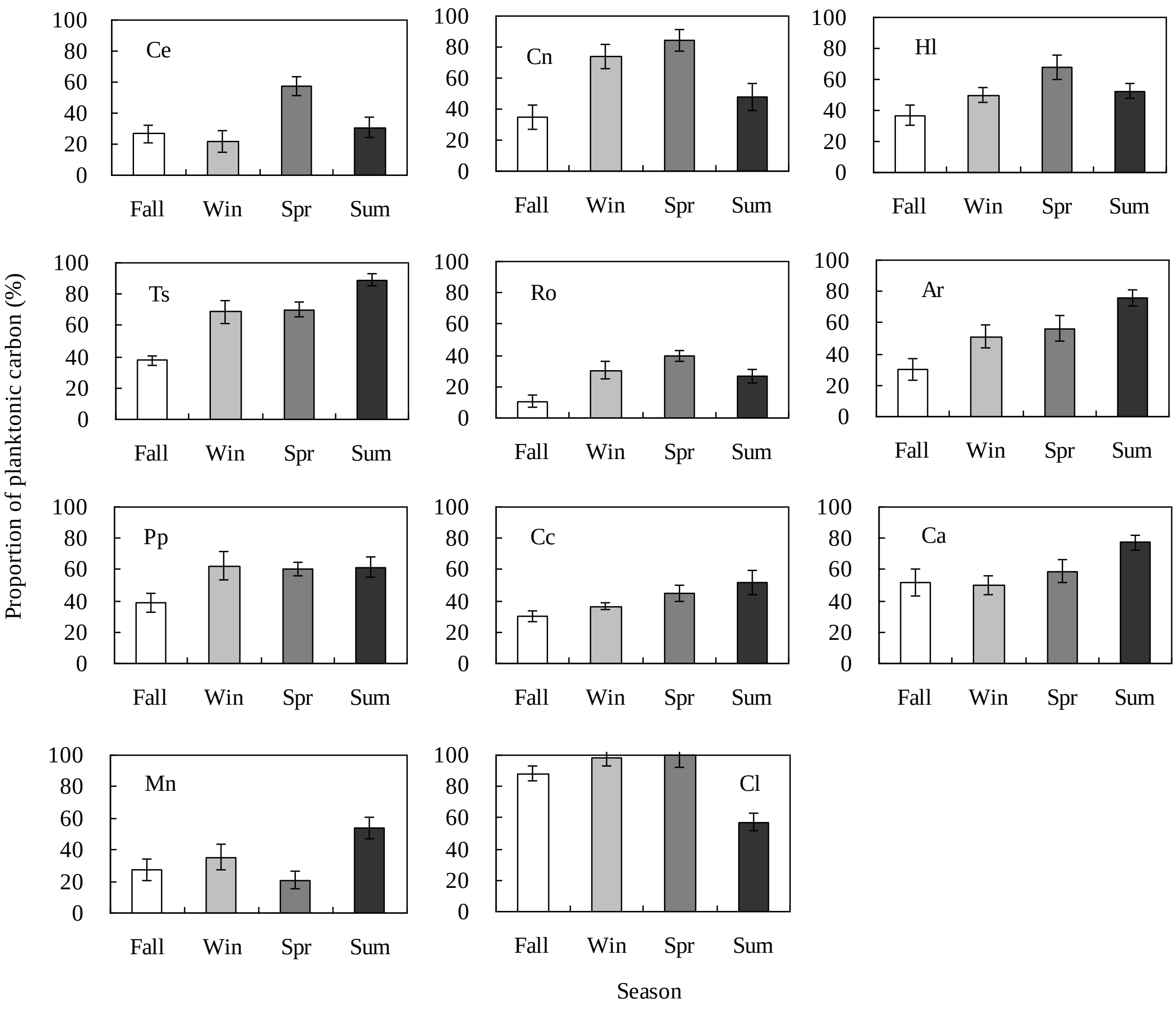

**FIGURE 3**. The proportional contribution of planktonic carbon to 11 consumer species in Meiliang Bay (Lake Taihu) in different seasons based on the two-source mixing model. Ce: *Cultrichthys erythropterus*; Cn: *Coilia nasus*; Hl: *Hemiculter leucisculus*; Ts: *Toxabramis swinhonis*; Ro: *Rhodeus ocellatus*; Ar: *Abbottina rivularis*; Pp: *Pseudorasbora parva*; Cc: *Cyprinus carpio*; Ca: *Carassius auratus*; Mn: shrimp *Macrobrachium nipponense*; Cl: Chironomid larvae. Fall, Win, Spr, and Sum in the diagram represent fall, winter, spring, and summer, respectively.

in food categories or food items in Meiliang Bay (Lake Taihu), suggesting that many fishes changed their diet seasonally. Furthermore, many researchers reported that seasonality in the stable isotope signatures of primary producers was transferred to higher trophic level consumers in eutrophic lakes[43,44,45,46]. Consequently, seasonal fluctuations in consumer $\delta^{13}$C and $\delta^{15}$N were probably attributed to the combined impacts of temporal variations in $\delta^{13}$C and $\delta^{15}$N of basal resources and the diet shift of fishes.

We initially expected to utilize phytoplankton and periphyton to assess the contributions of planktonic and benthic energy sources to littoral consumers. However, stable isotope signatures of phytoplankton and periphyton showed great temporal variations during this study (see Fig. 4), and thus were not suitable to evaluate the contribution of planktonic and benthic carbon sources of consumers. It has been documented that the freshwater snail *B. aeruginosa* fed mainly on periphyton[47], whereas the mussel *Cristaria plicata* primarily filtered phytoplankton from the water column[48]. Furthermore, in the present

**TABLE 4**
**Between-Quarter Differences in the Proportional Contributions of Planktonic Energy Sources to 11 Consumer Species Assessed by Two-Way ANOVA Analyses**

| Season | Proportional Contribution of Planktonic Carbon (%) | | | | | | | | | | | Similarity* |
|---|---|---|---|---|---|---|---|---|---|---|---|---|
| | Ce | Cn | Hl | Ts | Ro | Ar | Pp | Cc | Ca | Mn | Cl | |
| Fall | 26.6 | 34.9 | 36.9 | 37.8 | 10.4 | 30.1 | 38.9 | 30.0 | 51.6 | 27.0 | 88.3 | A |
| Winter | 21.8 | 74.0 | 49.9 | 68.7 | 30.5 | 51.2 | 62.3 | 36.6 | 50.2 | 35.1 | 98.1 | B |
| Spring | 57.4 | 84.3 | 68.0 | 70.1 | 39.5 | 56.3 | 60.1 | 44.6 | 58.8 | 20.8 | 100.0 | B |
| Summer | 30.7 | 48.1 | 52.4 | 88.9 | 26.8 | 75.8 | 61.5 | 52.0 | 77.4 | 53.7 | 57.1 | B |

Ce: *Cultrichthys erythropterus*; Cn: *Coilia nasus*; Hl: *Hemiculter leucisculus*; Ts: *Toxabramis swinhonis*; Ro: *Rhodeus ocellatus*; Ar: *Abbottina rivularis*; Pp: *Pseudorasbora parva*; Cc: *Cyprinus carpio*; Ca: *Carassius auratus*; Mn: shrimp *Macrobrachium nipponense*; Cl: Chironomid larvae.

* Different capital letters represent significant between-quarter differences ($p < 0.05$).

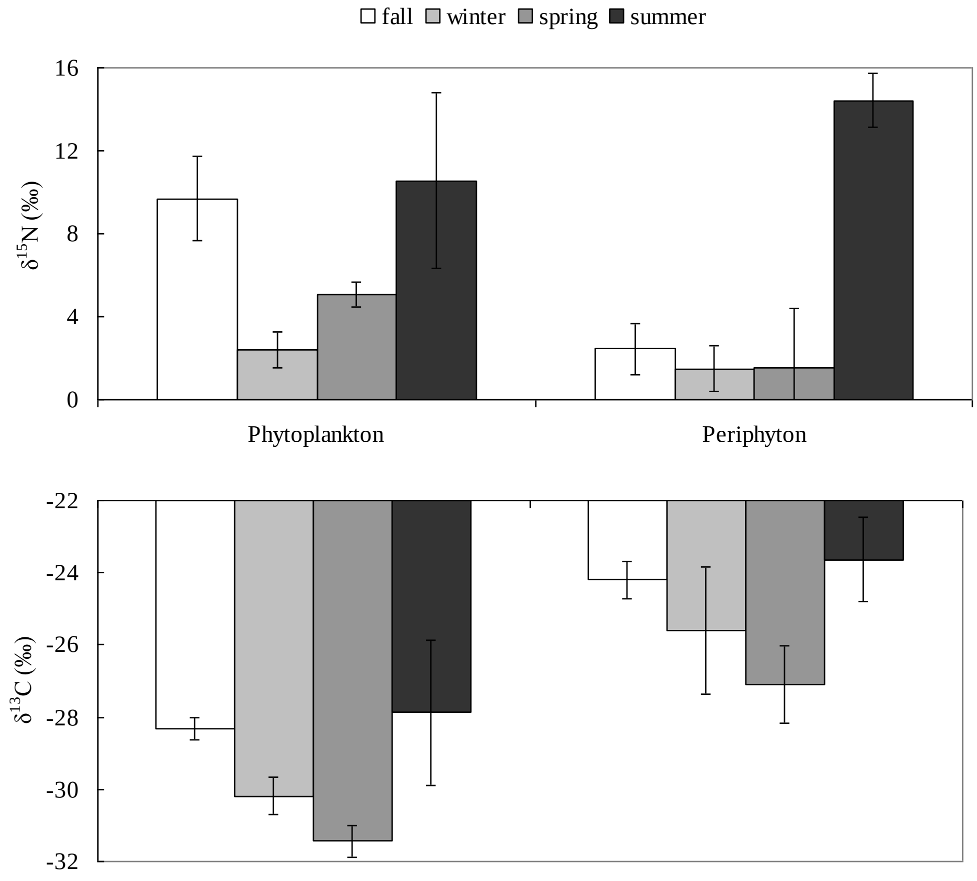

**FIGURE 4**. Stable isotope signatures (mean ± 1SD) of phytoplankton and periphyton in the littoral zones of Meiliang Bay (Lake Taihu) during different seasons. Data concerning phytoplankton were cited from Zhou et al.[18].

study, *B. aeruginosa* and *C. plicata* yielded lesser variation of isotopic signatures across seasons, implying that *B. aeruginosa* and *C. plicata* effectively integrated the temporal variation of $\delta^{13}C$ at the base of the littoral and pelagic food webs[23]. So, these two long-lived macroinvertebrates could reflect the phytoplankton- and periphyton-derived carbon sources, and served as baseline indicators (trophic position = 2) of planktonic and benthic energy sources[31,38,39].

The averaged $\delta^{13}C$ of *B. aeruginosa* showed 6.6‰ higher than that of *C. plicata*, suggesting that $\delta^{13}C$ in the base of the littoral food web was more enriched than that in the base of the pelagic food web, which was in agreement with the results of France[49]. Moreover, subtotal species situated between littoral and pelagic baselines for $\delta^{13}C$, apart from Chironomid larvae (in spring). Great differences between $\delta^{13}C$ of the littoral and pelagic baselines allowed for the application of the two-end-member mixing model in the present study.

Great differences between the $\delta^{13}C$ values of different consumers were indicated at each quarter, reflecting their utilization of carbon sources with clearly distinct isotope signatures. Since modification of $\delta^{13}C$ values during the foraging process was usually less than 1‰, animals that fed on the same food source presumably exhibited similar $\delta^{13}C$ values[50]. So, in the present study, consumer $\delta^{13}C$ exhibited differences of over three trophic positions at each quarter. However, for $\delta^{15}N$, differences of approximately two trophic positions between different consumers were found at each quarter, based on the generally assumed $\delta^{15}N$ enrichment of 3.4‰ per trophic level[23]. This reflected that littoral fishes and invertebrates depended on the energy source from coupling benthic and planktonic pathways. Even so, the predominant energy source for most species was highly variable during different seasons, suggesting a seasonal trophic shift of littoral consumers. Specifically, seasonality in energy mobilization of consumers focused on two aspects. First, the species number of consumers that relied mainly on planktonic carbon showed seasonal increase: fall < winter < spring or summer. In fall, two of the 11 species derived energy sources primarily from phytoplanktonic production, suggesting the dominance of benthic energy mobilization. In contrast, the species number of consumers increased to six in winter and indicated that planktonic carbon dominated the energy supply of consumers in winter. Furthermore, eight of the 11 species showed strong reliance on planktonic carbon in spring and summer. The seasonal pattern of the stepwise increase in species numbers of consumers concerning planktonic reliance was in accordance with the results of statistical analysis, which indicated statistically significant differences between fall and other seasons ($p < 0.05$). Stable isotope data suggested that settling phytoplankton was increasingly incorporated into littoral food webs along a gradient of oligotrophic to eutrophic lakes in Denmark[7]. Meiliang Bay is part of a eutrophic lake that resulted from anthropogenic activities (e.g., runoff of industrial wastewater) and indicated an algae-enriched aquatic ecosystem. In spring and summer, the water column in the littoral zone of Meiliang Bay showed remarkably high biomass of phytoplankton than those in fall and winter. High levels of phytoplankton production reduced light penetration to the sediments in eutrophic lakes, and thus limited periphyton and macrophyte productivity[21,51,52]. In this way, planktonic productivity greatly subsidized the littoral food web in warm seasons.

Second, most consumer species showed seasonal variation in the percentages of planktonic reliance and, moreover, these species were composed of carnivorous and omnivorous fishes. Top predators tracked changes in resource availability through their selection of prey and, hence, integrated food web processes over time[53]. In contrast with omnivorous fishes, carnivorous fishes shifted resource use twice. Carnivorous fishes would consume their preferred prey (i.e., other fishes) when available. However, when prey fishes became scarce due to intense predation pressure, carnivorous fishes would switch to feeding on benthic invertebrates[12,54]. Benthic resources maintained carnivorous populations during the times when their preferred prey were scarce[8]. Hence, compared with omnivorous fishes, energy mobilization of carnivorous fishes was more sensitive to resource availability.

The herbivorous fish *R. ocellatus* and omnivorous consumers *Carassius auratus* and Chironomid larvae did not change their dominant energy source during the study period, elucidating their specialization of resource use. Although biomass of phytoplankton changed seasonally, phytoplankton production was still enough to support higher trophic level predators because Meiliang Bay was an algal-enriched ecosystem[15,18.19]. Absolute reliance on phytoplankton-derived carbon for *C. auratus* and Chironomid larvae might be related to the predominance of phytoplankton biomass in Meiliang Bay. In contrast, benthic energy mobilization dominated the energy supply for *R. ocellatus* all along regardless of abundant algal biomass. Moreover, some consumer species utilized benthic resources as the main energy source at each quarter, suggesting the importance of a benthic energy source in supporting predators in the littoral zone of Meiliang Bay.

### ACKNOWLEDGMENTS

We gratefully acknowledge the field assistance of Dawen Zhang and Jialin Ye with sample collection. We also thank Taihu Lake Ecosystem Research Station, Chinese Academy of Sciences for providing physicochemical data. This research was partially supported by the National Basic Research Program of China (Grant No. 2008CB418001-1), the National Natural Science Foundation of China (Grant No. 30870428), and State Key Laboratory of Freshwater Ecology and Biotechnology of China (Grant No. 2011FB09).
